# MicroRNA-21 plays a role in exacerbating chronic obstructive pulmonary disease by regulating necroptosis and apoptosis in bronchial epithelial cells

**DOI:** 10.18332/tid/202182

**Published:** 2025-03-18

**Authors:** Zhengpeng Zeng, Xuelian Liu, Fei Xiang, Xue He, Jiahui Li, Hanying Liu, Lihua Xie

**Affiliations:** 1 Health Management Medicine Center, The Third Xiangya Hospital, Central South University, Changsha, China; 2 Respiratory and Critical Care Medicine, The Third Xiangya Hospital, Central South University, Changsha, China; 3 Changsha Kexin Cancer Hospital, Changsha, China

**Keywords:** cigarette smoke, necroptosis, apoptosis, microRNA-21, chronic obstructive pulmonary

## Abstract

**INTRODUCTION:**

Bronchial epithelial cell damage is an important determinant of the severity of chronic obstructive pulmonary (COPD). However, the exact molecular mechanisms underlying this cell death in COPD development are not well understood. This study investigates the involvement of microRNA-21 (miR-21/miRNA-21) in COPD and its underlying molecular mechanism.

**METHODS:**

A mouse model of COPD was created by exposing the mice to cigarette smoke (CS) and injecting them with cigarette smoke extract (CSE). Both wild-type mice and miR-21 knockout (miR-21-/-) mice were used to investigate the role of microRNA-21 (miR-21) in exacerbating COPD. Various assays and analyses were performed, including HE staining, tunel staining, enzyme-linked immunosorbent assay (ELISA), flow cytometry, quantitative real-time polymerase chain reaction (RT-qPCR), and western blotting (WB) to measure outcomes such as the pathological morphological changes, necroptosis, apoptosis, and levels of inflammatory factors.

**RESULTS:**

Our results revealed an upregulation of miR-21 in the lung tissue of COPD model mice. Additionally, knockout of miR-21 resulted in decreased levels of bronchial epithelial cell necroptosis and apoptosis, as evidenced by the downregulation of tumor necrosis factor receptor 1 (TNFR1), phosphoryl-mixed lineage kinase domain-like protein (p-MLKL) and caspase-3. This downregulation of necroptosis and apoptosis ultimately led to a reduction of inflammatory factors and damage-associated molecular patterns (DAMPs), such as tumor necrosis factor-α (TNF-α), interleukin-1β (IL- 1β), and interleukin-6 (IL-6) and high mobility group protein B1(HMGB1) in the lungs, thereby ameliorating COPD.

**CONCLUSIONS:**

Our findings suggest that miR-21 contributes to the worsening of chronic obstructive pulmonary disease by modulating necroptosis and apoptosis in bronchial epithelial cells, providing a new theoretical basis for the pathogenesis of chronic obstructive pulmonary disease.

## INTRODUCTION

Chronic obstructive pulmonary disease (COPD) is the most common respiratory system disease that seriously threatens health and is also the third leading cause of death globally, resulting in significant social, economic, and medical burdens^1^. COPD is a progressive disease associated with lung parenchymal damage and small airway lesions, characterized by persistent airflow limitation and impaired lung function^2^. Tobacco smoking remains the main risk factor for the incidence and mortality of COPD^3^. Inflammation plays an important role in the occurrence and development of COPD, such as the overexpression of inflammatory mediators and cytokines, and the activation of inflammatory signaling pathways^4,5^. However, the cellular and molecular mechanisms of the pathophysiology are still limited. Bronchial epithelial cells, as the first line of defense against cigarette smoke, can trigger immune and inflammatory processes by secreting various mediators such as cytokines, chemokines, and growth factors^6,7^. In summary, changes in bronchial epithelial cell damage play a key role in promoting the development of COPD, but the specific mechanisms involved still require further research. Therefore, it is critical to understand the molecular mechanisms of bronchial epithelial cell injury and excessive inflammation in the process of COPD.

MicroRNAs (miRs/miRNAs) are small non-coding RNAs that regulate proteins at the mRNA level. They range in length from 18 to 22 nucleotides and are widely present in eukaryotes^8-11^. Previous studies have reported abnormal expression of miR-21 in chronic obstructive pulmonary disease^8-11^. Changes in the levels of these miRNA have been shown to affect the expression of certain steps in key signaling pathways, including TNFR1-mediated necroptosis and caspase-3 in apoptosis^12,13^. Necroptosis, a unique type of programmed cell death distinct from apoptosis, is triggered by a series of death receptors such as TNFR1. Patients with severe COPD exhibited increased levels of MLKL protein in the epithelium and macrophages, as well as elevated levels of receptor-interacting protein kinase 3 (RIPK3) and MLKL in lung tissue. Deficiency of RIPK3 or MLKL could alleviate CS-induced airway inflammation, airway remodeling, and emphysema^14,15^. The necroptotic pathway is initiated by the activation of RIPK1, RIPK3, and mixed lineage MLKL through the interaction of the death receptor. This process ultimately leads to disruption of cell membrane integrity, release of DAMPs and inflammatory factors, thereby inducing more severe inflammation^16,17^. Apoptosis is a complex and multi-stage process involving many genes and also plays a role in the pathogenesis of COPD. The expression and function of TNF-α, caspase-3 in bronchial epithelial cell apoptosis in COPD have been extensively studied^18^. These genes appear to play important roles in the pathogenesis of diseases and can serve as important prognostic markers.

In this study, we constructed a mouse model of chronic obstructive pulmonary disease to investigate the mechanism by which miR-21 exacerbates COPD through the regulation of necroptosis and apoptosis in bronchial epithelial cells.

## METHODS

This is an animal model study aimed at exploring the role of miRNA-21 (miR-21) in exacerbating chronic obstructive pulmonary disease (COPD). Our team established a COPD model by exposing mice to cigarette smoke (CS) and injecting cigarette smoke extract (CSE), using C57BL/6 mice and miR-21 knockout (miR-21-/-) mice to compare the effects of miR-21 on the development of COPD. Various assays and analyses were conducted to measure outcomes such as necrotic apoptosis, cellular apoptosis, and levels of inflammatory factors.

### Animal and animal model establishment

Wild mice were purchased from Slack Corporation (Changsha, China), and miR-21-/- mice were obtained from the University of Texas Southwestern Medical Center; 8-week-old male (Wt: 22.28 ± 2.73 g) C57BL/6 and miR-21–/– mice were randomly divided into a control group and a COPD model group (n=5 per group). These animals were housed and maintained in the Experimental Animal Center of Xiangya Third Hospital, Central South University. All animals were kept in clean rooms with a temperature of 23–25°C, a humidity of 50–60%, and a 12-h light/dark cycle. We established a model of COPD by exposing the mice to cigarette smoke (CS) and intraperitoneal injection of cigarette smoke extract (CSE). The modeling box was made as previously described^19^. The total experimental period was 4 weeks. First, 5 cigarettes were burned simultaneously, and the smoke was generated for 15 minutes. Then, the box was opened, and the animals were allowed to rest for 5 minutes. This process was repeated, and it was considered as one cycle of CS exposure. Except on days 1, 12, and 23, the mice in the model group were exposed to 2 cycles of CS per day for a total of 28 days. On days 1, 12, and 23, the control group animals were intraperitoneally injected with 0.3 mL/20 g PBS, while the model group animals were intraperitoneally injected with 0.3 mL/20 g CSE-PBS.

The procedures involving animals conformed to the Guide for the Care and Use of Laboratory Animals published by the National Institutes of Health and have been approved by the Institutional Animal Research and Use Committee of Central South University. The animals received humane care and were euthanized by CO_2_ inhalation.

### Preparation of CSE

An unfiltered Furong cigarette (tar 13 mg, nicotine 1.0 mg, carbon monoxide 14 mg per cigarette; Hunan Tobacco Industry Co., Ltd., Changsha, China) was burned. One end of a sterile device containing 10 mL of PBS or RPMI-1640 medium was connected to a cigarette holder and the other end to a vacuum pump set at 0.1 kPa, until the cigarette was completely burned out. The solution was then passed through a 0.22 μm pore size filter (Fisher Scientific International, Hampton, NH, USA) to remove particles and bacteria, and used for intraperitoneal injection or cell intervention. The solution was freshly prepared for each injection. The collected stock was set to 100% and CSE was diluted at the desired concentration at the time of use for experiments. The amount of CSE was 2.5% and the intervention duration was 48 h.

### Cell culture and treatment

The 16HBE (human bronchial epithelial) cells were obtained from the Advanced Research Center of Central South University. The cells were maintained in RPMI-1640 medium (Life Technologies/Gibco, USA) with 5% CO_2_ and supplemented with 5% fetal bovine serum (FBS, Life Technologies/Gibco, USA). When the cell confluence reached about 70%, the cells in exponential growth phase were seeded into 6-well plates for experiments.

### Real-time quantitative PCR

The total RNA was extracted using Trizol reagent (Takara, DaLian, LiaoNing, China) according to the manufacturer’s protocol, and SYBR Green PCR Master Mix (Takara, DaLian, LiaoNing, China) was used to detect the mRNA levels. The expression of selected mRNA was quantified by two-step quantitative real-time PCR (Applied Biosystems, Carlsbad, California, USA). The relative expression levels were determined by applying the DD cycle threshold method using beta actin as an endogenous control.

### MicroRNA transfection

Cells in exponential growth phase were seeded at 5×104 cells/well in 6-well plates and incubated overnight. We added miR-21 mimic (Qiagen, Germany) (5 nM/5 μL) or miR-21 inhibitor (Germany) (50 nM/5 μL) to DMEM (90 μL), gently mixed, and incubated it at room temperature for 5 minutes. We added the transfection reagent (HiPerFect Transfection Reagent) to the above mixture, gently vortexed, and incubated at room temperature for 15 minutes. We added the above 100 μL to the wells containing 400 μL of culture medium, and gently mixed, then incubated in a cell culture incubator for 48 h before the experiment.

### Protein extraction and Western blot analysis

The cells and the homogenate of lung tissue were lysed in RIPA lysate for 30 min on ice. Bicinchoninic acid assay (BCA) protein quantitation kit (Wellbio Inc, Changsha, China) was used for protein measurement. Equal amounts of protein were separated by 12% sodium dodecyl sulfate-polyacrylamide gel electrophoresis (SDS-PAGE), transferred to polyvinylidene fluoride (PVDF) membranes, incubated with respective primary and secondary antibodies (TNFR1, p-MLKL, MLKL were obtained from Abcam, Caspase-3 and GAPDH were obtained from Proteintech), and detected using enhanced chemiluminescence. Protein bands on the blot were quantified using a ChemiDoc XRS+ chemiluminescence imaging system (Bio-Rad, Hercules, CA). The level of GAPDH was measured as a control for densitometry analysis.

### Histomorphology of lung tissue

The mouse was anesthetized with 1% pentobarbital sodium (60–70 mg/kg, i.p.), they were fixed on an animal dissecting table, and their chests were incised to expose the lung tissue. The left lung lobe was inflated with 4% paraformaldehyde under a constant pressure of 25 cm H_2_O and then fixed with 4% paraformaldehyde for 24 h. After paraffin sectioning of the lung tissue, hematoxylin and eosin staining experiment was performed. The emphysema was quantified by measuring the mean linear intercept (MLI), mean alveolar septal thickness (MAST), and destructive index (DI).

### Tunel staining

Tunel assay was performed on paraffin sections using the kit according to the manufacturer’s instructions (Nanjing Keki TUNEL, KGA704). The cell nuclei were stained with DAPI. Images were captured under a fluorescence microscope (Motic, BA410T). The positive labeled cells were counted using ImageJ software.

### Cell apoptosis assay

Cell apoptosis of the 16HBE cells was detected with the flow cytometry method (FCM) using an AnnexinVPI Apoptosis Detection Kit (BD, Cambridge, UK). Briefly, the cells were collected, washed with PBS, and suspended in 500 μL of binding buffer. The cells were incubated with AnnexinV and PropidiumIodide (PI) at room temperature for 10 min. The relative quantitative apoptosis was analyzed using FCM.

### Statistical analysis

Data analysis was performed using GraphPad Prism version 6.01 (GraphPad Software, San Diego, CA), with results presented as mean ± SD from independent replicates. The differences between the two means were assessed through an unpaired Student’s t-test (two tails). For comparisons involving multiple groups, one-way or two-way ANOVA was utilized, followed by Bonferroni’s *post hoc* test. Values of p<0.05 were regarded as statistically significant.

## RESULTS

**Figure 1:**
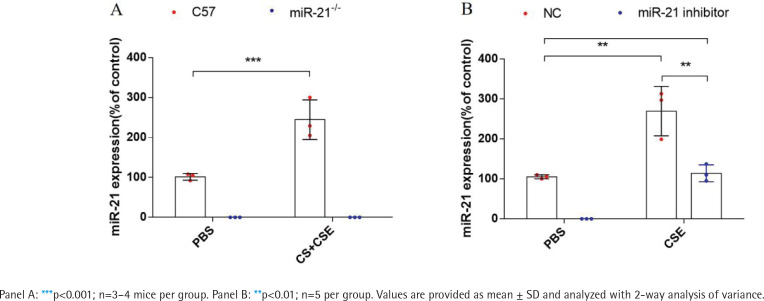
MiR-21 was up-regulated in the lung tissue of COPD model mice and the 16HBE cells treated with CSE: A) RT-qPCR detection of miR-21 expression in the lung tissue of COPD mouse model. Control group: C57/miR-21–/–+PBS. C57+CS+CSE group: C57BL/6 mice were intervened by CS exposure combined with CSE intraperitoneal injection. miR-21-/-+ CS +CSE group: miR-21–/– mice were intervened by CS exposure combined with CSE intraperitoneal injection; B) 16HBE cells were transfected with miR-21 inhibitor before CSE treatment. The levels of miR-21 were determined by RT-qPCR assays

MiR-21 was up-regulated in the lung tissue of COPD model mice and the 16HBE cells treated with CSE. We used the RT-qPCR method to detect the expression of miR-21 in the lung tissue of COPD model mice and 16HBE cells intervened by CSE. The expression level of miR-21 in the lung tissue of COPD model mice and 16HBE cells intervened by CSE was significantly higher than that in the control group (Figures 1A and 1B). Nevertheless, whether or not in the COPD model mice, miR-21 was almost not expressed in the lung tissue of miR-21-/- mice. Additionally, after intervention with the miR-21 inhibitor, the expression of miR-21 in CSE-induced 16HBE cells is partially inhibited (Figures 1A and 1B).

### miR-21 exacerbates emphysema in COPD mice

**Figure 2:**
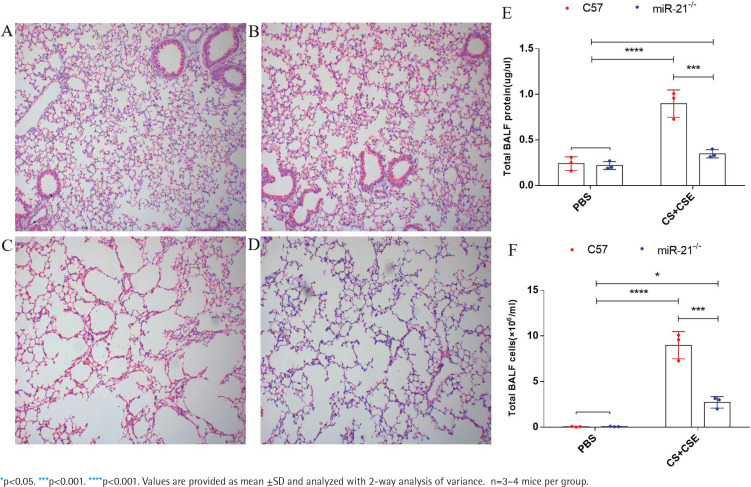
miR-21 exacerbates emphysema in COPD mice: A–D) The pathological morphological changes of lung tissue after knocking out miR-21 while using the same method to build the COPD model; A, B) Control group: C57/miR-21–/–+PBS; C) C57+CS+CSE group: C57BL/6 mice were intervened by CS exposure combined with CSE intraperitoneal injection. HE staining of lung tissue from model mice (200×) showing an enlarged alveolar space, a thinner alveolar septum and a destroyed alveolar wall; D) miR-21-/-+ CS +CSE group: miR-21–/– mice were intervened by CS exposure combined with CSE intraperitoneal injection. HE staining of lung tissue from miR-21-/- mice (200×) showing increased alveolar septum thickness, reduced alveolar size, and destruction of the alveolar wall; E) Total protein concentration in the BALF; F) Total cells in the BALF

To further explore the role of miR-21 in mouse COPD, we used miR-21-/- mice constructed a COPD model and observed the emphysema status. HE staining showed that lung tissue in the C57+CS+CSE group had significant morphological damage, including inflammatory cell infiltration and aggravated emphysema, while this damage was significantly reduced in the miR-21-/-+CS+CSE group (Figures 2C and 2D). miR-21-/- treatment partially reversed the inflammatory cell infiltration and emphysema induced by cigarette smoke (Figure 2D). Cigarette smoke increased the levels of total protein and total cell count in BALF, while miR-21/-treatment reduced their levels (Figures 2E and 2F), indicating that reducing the expression of miR-21 could significantly improve emphysema in COPD mice.

### Knockout of miR-21 reduces lung inflammation in COPD mice

**Figure 3:**
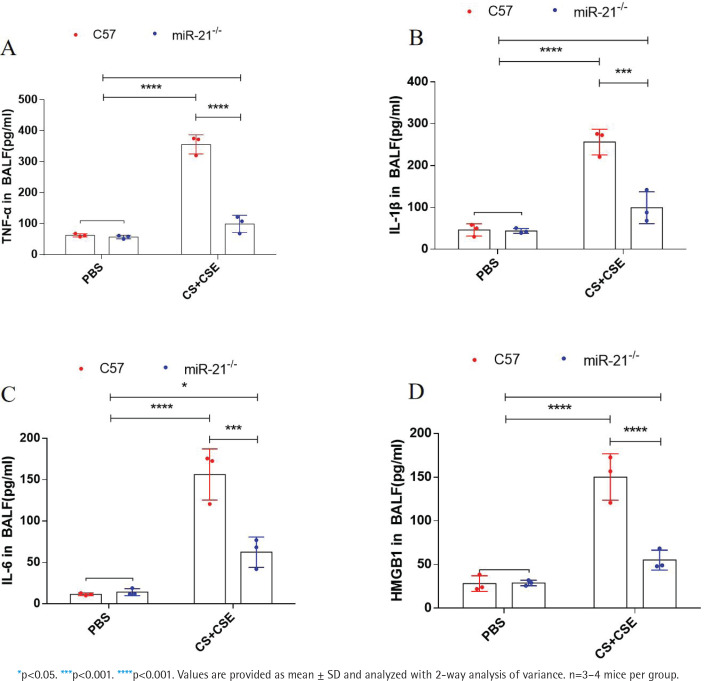
ELISA assays were performed to measure the levels of TNF-α, IL-1β, IL-6 and HMGB1 in the BALF after CS+CSE or combined miR-21-/- treatment (A–D). Control group: C57/miR- 21–/–+PBS. C57+CS+CSE group: C57BL/6 mice were intervened by CS exposure combined with CSE intraperitoneal injection. miR-21-/-+ CS +CSE group: miR-21–/– mice were intervened by CS exposure combined with CSE intraperitoneal injection

We used ELISA kits to detect the levels of TNF-α, IL-1β, IL-6, and HMGBI in BALF. The results showed that the levels of TNF-α, IL-1β, IL-6, and HMGBI were significantly upregulated in the C57+CS+CSE group. miR-21-/- treatment reduced the levels of inflammatory factors in BALF (Figures 3A–3D). This result suggests that miR-21-/- improves emphysema in COPD mice by inhibiting their inflammatory levels.

### miR-21 targets TNFR1 to regulate necroptosis and apoptosis in COPD mice

We found that the protein level of TNFR1 in lung tissue of the C57+CS+CSE group was significantly increased, while miR-21-/-+CS+CSE treatment reduced the expression of TNFR1 (Supplementary file Figure 1A). In addition, we detected the expression of caspase-3 (one of the apoptosis execution proteins) and p-MLKL (cell necrosis execution protein), markers of necroptosis and apoptosis, respectively. The results showed that cigarette smoke stimulation increased the expression of caspase-3 and p-MLKL in lung tissue, while miR-21-/- treatment downregulated the expression of these proteins (Supplementary file Figure 1A). Furthermore, miR-21-/- treatment reduced cigarette smoke-induced apoptosis in lung tissue (Supplementary file Figure 1B). These data indicate that miR-21 targets TNFR1 to promote necroptosis and apoptosis in lung tissue of COPD model induced by cigarette smoke.

### miR-21 targets TNFR1 to accrete necroptosis and apoptosis in 16HBE cells

We found that the protein level of TNFR1 in 16HBE cells of the CSE group was significantly increased, while the miR-21 inhibitor treatment reduced the expression of TNFR1 (Supplementary file Figure 2A). In addition, the results showed that CSE stimulation increased the expression of caspase-3 and p-MLKL in 16HBE cells, while miR-21 inhibition downregulated the expression of these proteins (Supplementary file Figures 2D–2E). Furthermore, inhibition of miR-21 treatment reduced CSE-induced apoptosis in 16HBE cells (Supplementary file Figure 2E). These data indicated that miR-21 targets TNFR1 to inhibit necroptosis and apoptosis in 16HBE cells.

## DISCUSSION

We have confirmed that reducing miR-21 can alleviate necrosis and apoptosis by inhibiting TNFR1, playing an important role in the occurrence and development of COPD. Our results show that inhibiting miR-21 reduces the levels of inflammatory factors and DAMPS in lung tissue and improves emphysema in COPD mice. We found that miR-21 expression was significantly increased in COPD model mice, and TNFR1 expression was significantly decreased when miR-21 was knocked out, confirming that miR-21 can regulate TNFR1 expression. Similarly, a previous study showed that miR-21 upregulates TNF-α mRNA and protein expression levels through an unknown target in HeLa cells in cervical cancer, thereby affecting its proliferation. When TNF-α is upregulated by miR-21, it binds and activates the TNFR1 receptor in HeLa cells, the apoptosis program is activated^20^.

Our experimental results also indicate that the expression of key protein p-MLKL, closely related to necroptosis, is significantly increased in the lung tissue of COPD model mice. In addition, the expression of inflammatory factors such as TNF-α, IL-6, and DAMPs such as HMGB1 is significantly increased. Knocking out miR-21 can significantly reduce the occurrence of necroptosis, thereby reducing the levels of the aforementioned inflammatory factors and DAMPs. Necroptosis plays an important role in the occurrence and development of COPD^21,22^. Unlike apoptosis, necroptosis is triggered by a series of death receptors, such as tumor necrosis factor receptor 1 (TNFR1) and TNFR2 ^23,24^. The necroptosis pathway is activated by three key downstream mediators, namely RIPK1, RIPK3, and MLKL^13,25,26^. The entire process ultimately leads to the disruption of cell membrane integrity and the swelling of organelles, and the cell contents are released as DAMPs^27-29^. Due to necroptosis, DAMPs bind to various molecules on other cells in the interstitium, leading to the production of more inflammatory factors in other cells, resulting in more severe inflammation^30^. Our study suggests that inhibiting miR-21 can reduce the expression of p-MLKL by TNFR1, thereby reducing the level of inflammation in the COPD model. Moreover, according to the results of WB and tunel staining, the level of apoptosis in the lung tissue of COPD model mice was significantly higher than that of the control group, while miR-21-/- treatment reversed this phenomenon significantly. Of note, as shown in Supplementary file Figure 1B, these apoptotic cells are mainly bronchial epithelial cells, whose main function is to serve as a defense barrier, helping to maintain normal airway function.

Bronchial epithelial cells form the interface between the external and internal environments, making them the main target of cigarette smoke inhalation damage^6,31^.

The damage or death of bronchial epithelial cells is a key feature in the progression of COPD^32^. These bronchial epithelial cells can recruit and activate inflammatory cells by releasing chemotactic factors and cytokines, thereby initiating and coordinating immune and inflammatory responses, leading to chronic pulmonary inflammation and tissue damage^33^. Our research has found that CSE stimulation upregulates the expression of p-MLKL and caspase-3 in bronchial epithelial cells. Treatment with miR-21 inhibitor downregulates the expression of these molecules. Additionally, flow cytometry results showed that the inhibition of miR-21 expression significantly reduced the 16HBE cells’ apoptotic level under CSE intervention. Therefore, we speculate that inhibiting miR-21 may suppress bronchial epithelial cell necroptosis and apoptosis by targeting TNFR1.

### Limitations

Our research has certain limitations. This study primarily focuses on the role of miR-21 in necroptosis and apoptosis in a mouse model of COPD. COPD is a multifactorial disease, and other pathways and factors may also play significant roles, which were not explored in this study. Furthermore, this study mainly concentrates on the impact of miR-21 on COPD at specific time points, lacking an exploration of longitudinal data. Additionally, this research was conducted in a highly controlled laboratory environment, which may not accurately reflect the variability and complexity of human exposure to cigarette smoke and other environmental factors in the real world. Due to species-specific differences, findings in mice may not fully replicate the complexity of COPD in humans, and results obtained from animal models may not always translate directly to humans.

## CONCLUSIONS

Our research indicates that miR-21 is notably elevated in the lung tissues of COPD model mice and 16HBE cells exposed to cigarette smoke extract. The knockout of miR-21 in these mice led to decreased necroptosis and apoptosis of bronchial epithelial cells, as demonstrated by the downregulation of TNFR1, p-MLKL, and caspase-3. This reduction in cell death correlated with lower levels of inflammatory mediators and DAMPs, including TNF-α, IL-1β, IL-6, and HMGB1, which ultimately improved COPD symptoms in the model mice. These results imply that miR-21 is crucial in worsening COPD by facilitating necroptosis and apoptosis in bronchial epithelial cells, and inhibiting it could help reduce lung inflammation and tissue injury in COPD.

## Supplementary Material



## Data Availability

The data supporting this research are available from the authors on reasonable request.
