# Supplementary material for: MicroRNA-21 plays a role in exacerbating chronic obstructive pulmonary disease by regulating necroptosis and apoptosis in bronchial epithelial cells
Source: Tob Induc Dis. 2025 Mar 18;23:10.18332/tid/202182. doi: 10.18332/tid/202182 (PMC11915094; doi:10.18332/tid/202182)
Supplement: Supplementary file 1 [file TID-23-32-s1.pdf]

Supplementary File

Supplementary Figure 1

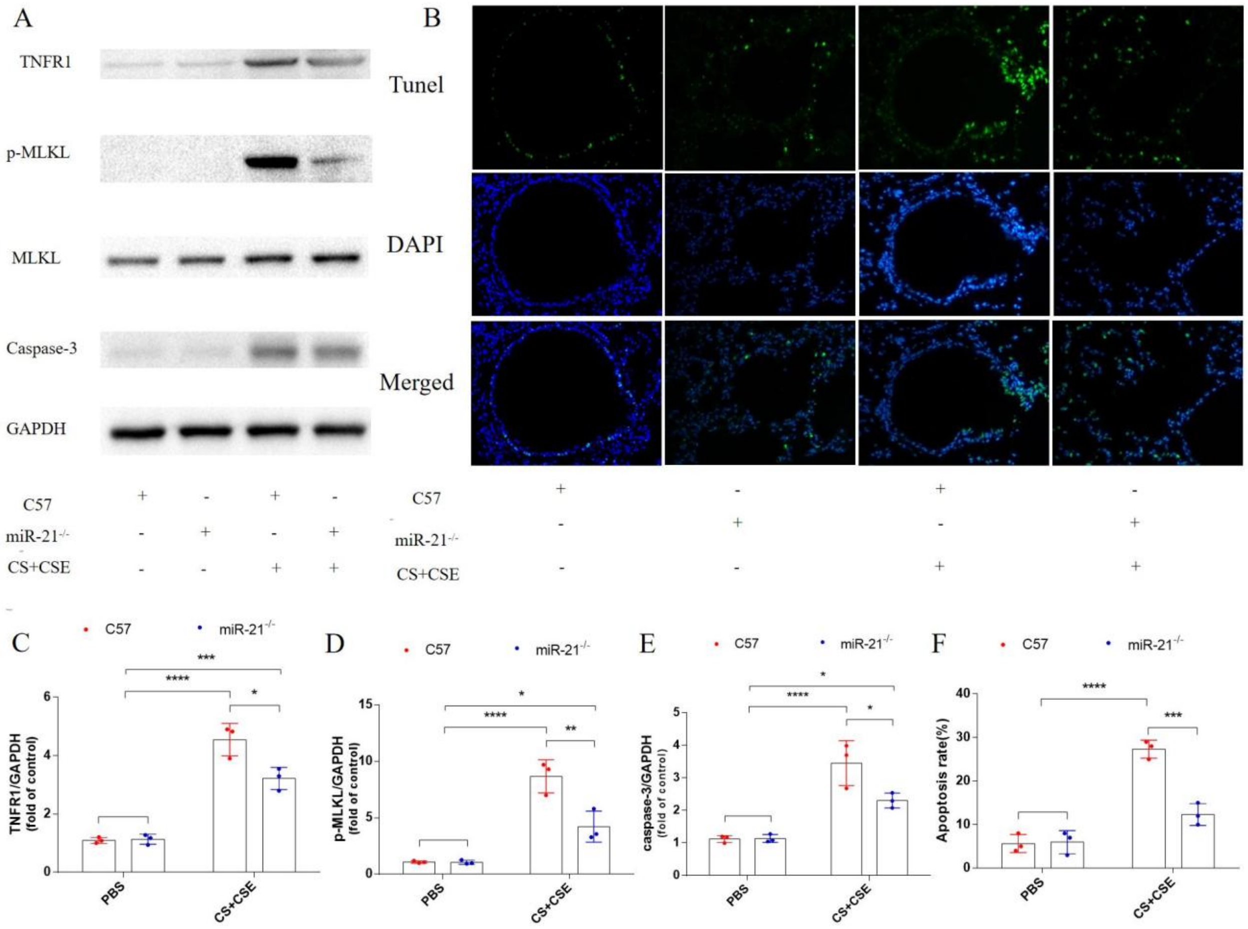

Figure 1. miR-21 targets TNFR1 to accrete necroptosis and apoptosis in COPD mice. Control group: C57/miR-21–/–+PBS. C57+CS+CSE group: C57BL/6 mice were intervened by CS exposure combined with CSE intraperitoneal injection. miR-21-/-+ CS +CSE group: miR-21–/– mice were intervened by CS exposure combined with CSE intraperitoneal injection. (A) Western blot analysis of tumor necrosis factor receptor 1 (TNFR1), phosphoryl -ated mixed lineage kinase domain like protein (p-MLKL), MLKL and caspase-3 in lungs of C57 mice and miR-21–/– mice with glyceraldehyde-3- phosphate dehydrogenase (GAPDH) as the loading control. (B) Apoptosis detected via TUNEL assay. (400×magnification). (F) Apoptosis rate in each group. n=3-4 mice per group. *P < 0.05, **P < 0.01, ***P < 0.001, ****P < 0.001. Values are provided as mean ± SD and analyzed with 2-way analysis of variance.

Supplementary Figure 2

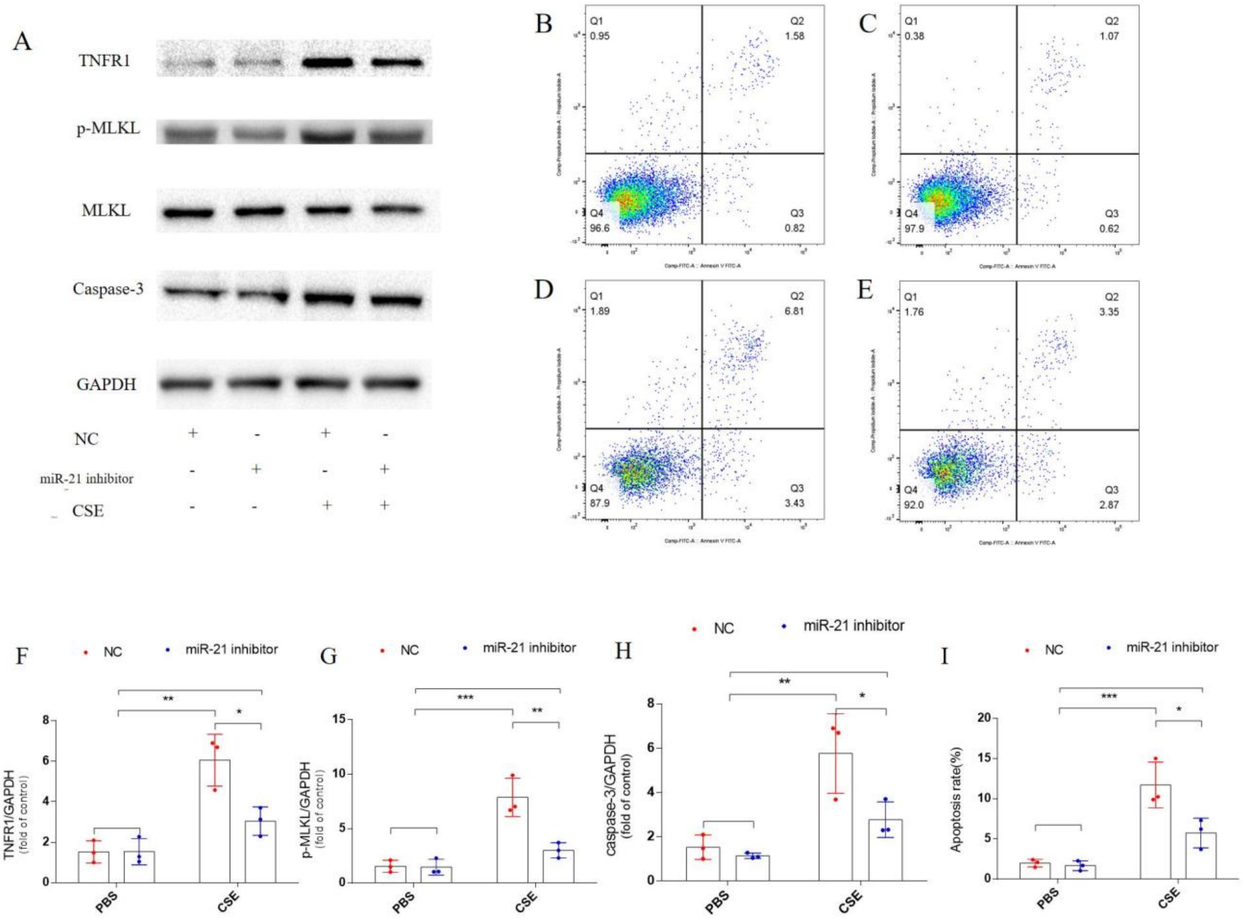

Figure 2. miR-21 targets TNFR1 to accrete necroptosis and apoptosis in 16HBE cells. 16HBE cells were transfected with miR-21 inhibitor before CSE treatment. (A) Western blot analysis of TNFR1, p-MLKL, MLKL, and caspase-3 in 16HBE cells or combined miR-21 inhibitor treatment with GAPDH as the loading control. (B-E) Cell apoptosis was measured by flow cytometry. B.NC+PBS, C. miR-21 inhibitor+PBS, D. NC+CSE, E. miR-21 inhibitor + CSE. n=5 per group. *P < 0.05, **P < 0.01, ***P < 0.001. Values are provided as mean ± SD and analyzed with 2-way analysis of variance.

© 2025 Zeng Z. et al.
